# Feeding hempseed cake alters the bovine gut, respiratory and reproductive microbiota

**DOI:** 10.1038/s41598-023-35241-1

**Published:** 2023-05-19

**Authors:** Thomas M. Winders, Devin B. Holman, Kaycie N. Schmidt, Sarah M. Luecke, David J. Smith, Bryan W. Neville, Carl R. Dahlen, Kendall C. Swanson, Samat Amat

**Affiliations:** 1 Department of Animal Sciences, North Dakota State University, Fargo, ND 58108-6050 USA; 2 Lacombe Research and Development Centre, Agriculture and Agri-Food Canada, 6000 C & E Trail, Lacombe, AB T4L 1W1 Canada; 3 Department of Microbiological Sciences, North Dakota State University, Fargo, ND 58108-6050 USA; 4 USDA ARS, Edward T. Schafer Agricultural Research Center, Fargo, ND 58102 USA; 5 USDA-ARS, U.S. Meat Animal Research Center, Clay Center, NE 68933 USA; 6 Center for Nutrition and Pregnancy, North Dakota State University, Fargo, ND 58108-6050 USA

**Keywords:** Microbiology, Microbial communities, Microbiome

## Abstract

A growing number of studies have investigated the feasibility of utilizing hemp by-products as livestock feedstuffs; however, their impact on livestock microbiomes remains unexplored. Here, we evaluated the effects of feeding hempseed cake on the gastrointestinal, respiratory, and reproductive microbiota in beef heifers. Angus-crossbred heifers (19-months old, initial body weight = 494 ± 10 kg [SE]) were fed a corn-based finishing diet containing 20% hempseed cake as a substitute for 20% corn dried distillers’ grains with solubles (DM basis; Control; n = 16/group) for 111 days until slaughter. Ruminal fluid and deep nasopharyngeal swabs (days 0, 7, 42, 70 and 98), and vaginal and uterine swabs (at slaughter) were collected, and the microbiota assessed using 16S rRNA gene sequencing. Diet affected the community structure of the ruminal (d 7−98; 0.06 ≤ R^2^ ≤ 0.12; *P* < 0.05), nasopharyngeal (d 98; R^2^ = 0.18; *P* < 0.001), and vaginal (R^2^ = 0.06; *P* < 0.01) microbiota. Heifers fed hempseed cake had increased microbial diversity in the rumen, reduced microbial richness in the vagina, and greater microbial diversity and richness in the uterus. In addition to the distinct microbial communities in the rumen, nasopharynx, vagina and uterus, we identified 28 core taxa that were shared (≥ 60% of all samples) across these sampling locations. Feeding hempseed cake appeared to alter the bovine gut, respiratory and reproductive microbiota. Our results suggest that future research aiming to evaluate the use of hemp by-products in livestock diet should consider their impact on animal microbiome and microbiome mediated animal health and reproductive efficiency. Our findings also highlight the need for research evaluating the impact of hemp-associated food and personal care products on the human microbiome.

## Introduction

To increase animal productivity in a sustainable manner, the livestock industry seeks to enhance feed efficiency and explore alternative and novel feeds^1^. The quest to identify low-cost and underutilized feed alternatives is also driven by an increase in costs of traditional feed sources and a rise in competition between livestock and humans for food crops^1^. Local alternative feeds such as co-products from ethanol production (corn and wheat distillers grains)^2,3^ and by-products of oilseed crops (soybean and canola meals, and cotton seed hulls)^4–6^ have been demonstrated to be viable feed alternatives. In recent years, there has been growing interest in exploring the feasibility of feeding industrial hempseed and its by-products to cattle^7–9^, sheep^10^, goats^11^ pigs^12^, and poultry^13^. The renewed interest in using industrial hemp by-products as alternative feed ingredients is due to (1) the legalization of industrial hemp cultivation in many parts of the world; and (2) the increasing global demand for industrial hemp from the food and beverage, personal care, and animal care industries^7^.

The seed of industrial hemp has high nutritive concentrations (protein, lipid, mineral and vitamins) and is also rich in antioxidants and bioactive compounds, that together make it appealing for use in functional foods and human medicine^7,14^. Oil extracted from hempseed contains large amounts of polyunsaturated fatty acids, which are known for their protective effects against cardiovascular diseases, cancer, and inflammatory conditions^15^. Two essential fatty acids, linoleic acid (18:2 omega-6) and alpha-linolenic acid (18:3 omega-3), are contained in greater abundance in hempseed oil compared to other vegetable oils^15^. In addition, the antioxidant (e.g. tocopherols and tocotrienols)^15,16^, anti-inflammatory^17^ and antimicrobial (e.g. volatile terpenes)^18,19^ properties of hempseed oil make it appealing to the functional food and pharmaceutical industries^14,15^.

Hempseed oil is also used in paint, detergent, varnish and other coating formulations (Fike, 2016), and the demand for hempseed oil is expected to increase^20^. Hempseed oil extraction creates a by-product known as hempseed cake (also sometimes termed hempseed meal) that contains high concentrations of nutritionally valuable fiber (50%), crude protein (30%) and oil (7%)^9,21^. Not surprisingly, there is growing interest in its potential use as a livestock feedstuff. Despite hemp’s nutritive and potential therapeutic values, incorporation of hemp by-products into animal diets has been restricted by regulatory authorities. For example, inclusion of hempseed cake in European ruminant diets must be less than < 50 g/kg (on a DM basis)^22^, but in the United States, neither hemp nor its by-products may be fed to livestock without FDA approval^23^. Similarly, the use of hemp products as livestock feed ingredients is restricted in Canada, and each hemp product requires government approval (RG-1 Regulatory Guidance) prior to use in livestock feed. These restrictions are due to concerns regarding the possible accumulation of cannabinoids, such as THC and cannabidiol (CBD), in edible tissues of animals fed hemp products^7,22–24^.

Whilst feeding hempseed by-products has been evaluated in several livestock species, the focus of these studies has been largely limited to the nutritional value of the by-products, that is digestibility and animal performance metrics. The impact(s) of feeding hempseed by-products on the animal microbiome is largely unexplored. The microbial communities residing within the gastrointestinal, respiratory and reproductive tracts are vital to animal health and productivity not only for their involvement in nutrient metabolism but also because they influence infectious and metabolic diseases^25–27^. Given the bioactive, anti-inflammatory, antimicrobial and anti-nutrient compounds present in hempseed by-products, we hypothesize that inclusion of hempseed by-products in cattle rations can induce alterations in the gut ecological and functional microenvironment, and that the impact of long-term ingestion of hempseed by-products may extend beyond the gut and may impact the respiratory and reproductive tract microbiota. To test this hypothesis, we conducted a longitudinal beef cattle experiment. The primary objective of this study was to evaluate the effects of feeding hempseed cake on the gastrointestinal, respiratory, and reproductive microbiota in finishing beef heifers. The secondary objective was to compare microbial communities associated with ruminal fluid, nasopharynx, vagina, and uterus, and to identify core bacterial taxa that are shared across these microbial ecosystems. Cattle have similar physiological traits (singleton pregnancy and gestation period) to humans and their microbiota are biogeographically and phylogenetically more similar to that of humans compared to rodents^28^. Therefore, coupled with the growing use of hemp and cannabidiol products as functional food and pharmaceutics, the findings reported here are not only important for cattle, but they also provide important speculative insights on the relationship between hemp product consumption and the human microbiome.

## Methods

All animal care and management practices were approved by the North Dakota State University Institutional Animal Care and Use Committee (Approved IACUC protocol# A21010). We confirm that all methods were carried out in accordance with relevant guidelines and regulations.

### Animal husbandry, experimental design, and dietary treatments

**Figure 1:**
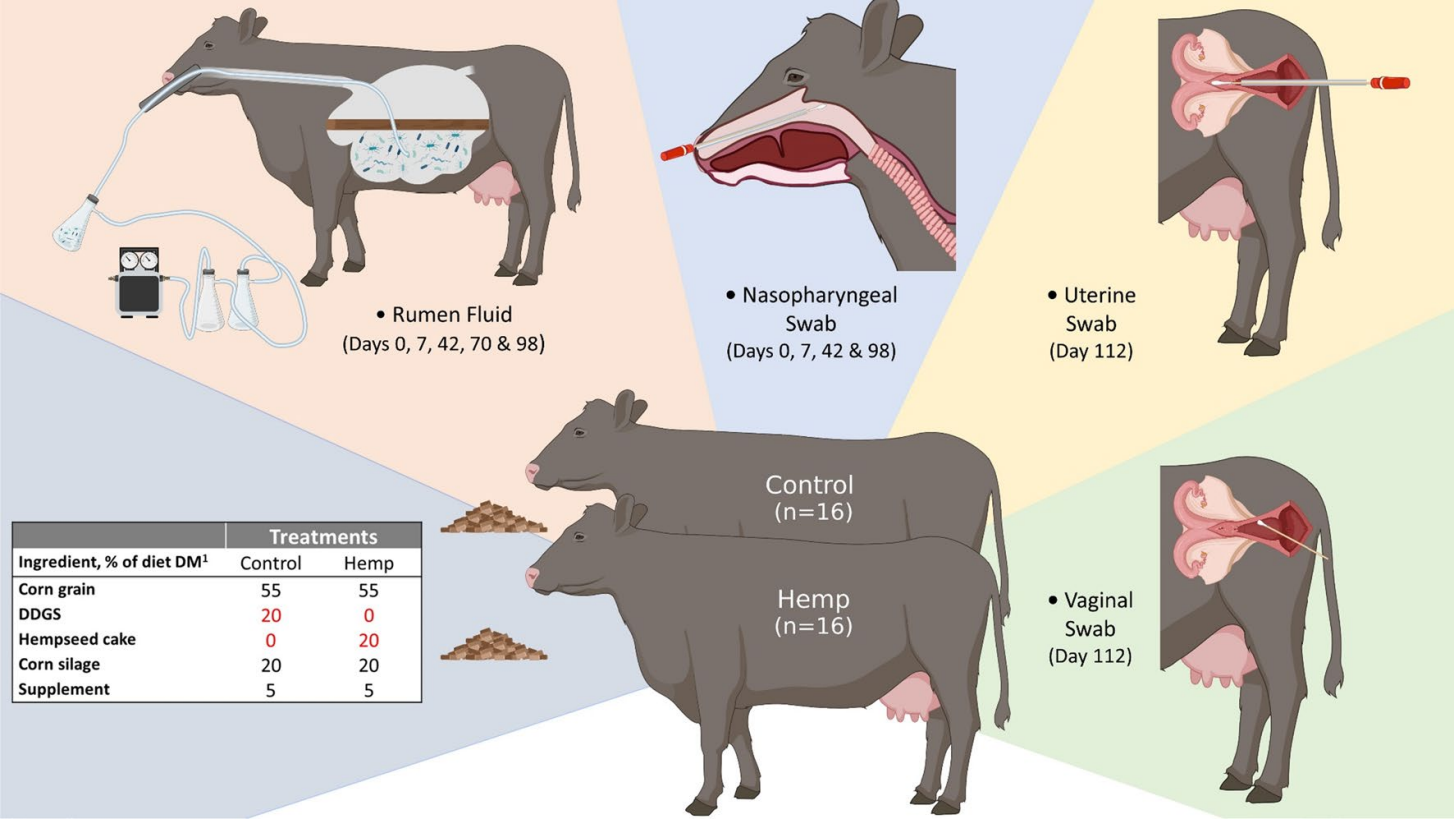
Study design, diets, and sampling regimen. This figure was created using Biorender.

A longitudinal (16-week long) study was conducted to evaluate the effect of hempseed cake inclusion in finishing diets on growth performance, carcass quality characteristics, plasma, urine, and tissue cannabinoid residues, feeding behaviour, and the gut, respiratory and reproductive tract microbiota of beef cattle. The live phase of the feeding study has been described in detail by Winders et al.^29^. Briefly, a total of 32 crossbred finishing heifers (initial body weight = 494 ± 10 kg [SE], average age = 19 months) were randomly assigned into either hemp (n = 16) or control (n = 16) groups (Fig. 1). The hemp group heifers received a formulated ration containing 20% hempseed cake (dry matter basis), whereas heifers in the control group received the same diet except that the hempseed cake was substituted with 20% corn dried distillers’ grains with solubles (DDGS). Corn DDGS are the most common ethanol by-product included in finishing beef cattle diets in the U.S.^30^ and are similar in nutrient composition to hempseed cake. Therefore, a diet containing 20% corn DDGS was used as the control diet. The two groups of heifers were housed in separate pens at the NDSU Beef Cattle Research Complex in Fargo (North Dakota, USA), and were individually fed the treatment diets for 111 days using the Insentec BV feeding system (Hokofarm Group, Marknesse, The Netherlands) which feed intake data for individual animals. The remaining 80% of the total mixed ration included 55% dry-rolled corn, 20% corn silage and 5% supplement (dry matter basis; Fig. 1).

### Ruminal fluid, nasopharyngeal, vaginal, and uterine sampling

Ruminal fluid and nasopharyngeal swab samples were collected across sampling days and heifer by the same personnel during the live phase portion of the study (days 0–98 days). Within a collection day, all samples were collected within a 3-h window (8 a.m.–11 a.m.). Vaginal and uterine swabs were collected at slaughter (days 112–120).

### Ruminal fluid sampling

Rumen fluid was collected on days 0, 7, 42, 70 and 98 as described by Amat et al.^31^. Briefly, a rigid metal speculum was placed into the mouth of the heifer and a flexible PVC stomach tube with multiple holes at the distal tip was passed through the speculum and into the esophagus (Fig. 1). The speculum was used to ensure that the plastic tube was not damaged by the heifer’s teeth and that the tube entered the esophagus and could be passed into the rumen and below the ruminal mat. A light vacuum was applied to the collection tube through which ruminal fluid (120 mL maximum volume) was aspired into a side-arm Erlenmeyer flask (Fig. 1). Separate tubing and collection flasks were used for each heifer to avoid cross-contamination. After thorough mixing, an aliquot of 40 mL of ruminal fluid was placed into a 50-mL falcon tube and immediately frozen on dry ice.

### Nasopharyngeal sampling

Deep nasopharyngeal swabs were collected on days 0, 7, 40 and 98 as previously described^31^ (Fig. 1). Briefly, prior to swab insertion, the right nostril of the heifer was wiped clean with 70% ethanol and a paper towel. An extended guarded swab (27 cm) with a rayon bud (MW 128, Medical Wire & Equipment, Corsham, England) was passed into the nostril and when the sheathed swab reached the nasopharynx area, the swab tip was advanced a few centimeters to swab the nasopharynx and rotated. The swab was withdrawn into the sheath and then removed from the nasal cavity. The tip of the swab (approx. 2.5 cm) was then snipped into a sterile microfuge tube using a sterilized wire cutter, and transported to the lab on ice. Upon arrival in the lab, nasopharyngeal swabs were transferred into 1 mL of brain heart infusion (BHI) broth containing 20% glycerol and stored at − 80 °C until DNA extraction.

### Vaginal and uterine sampling

Uterine and vaginal swabs were collected immediately after euthanasia upon the completion of the 111-day hempseed cake feeding trial for carcass data collection. Heifers were slaughtered on study days 112–120 (withdrawal days 0, 1, 4, and 8 as described by^29^. Pre-slaughter withdrawal periods were established to examine the depletion of cannabinoid residues from cattle tissues. Immediately upon euthanasia vaginal swabs were collected thoroughly cleansing the vulva with a paper towel saturated with 70% ethanol. The labia majora were held open with a gloved hand allowing the passage of a 15-cm sterile cotton-tipped swab (Puritan; Guilford, ME). When the swab tip reached the midpoint of the vagina, it was placed against the vaginal wall, swirled four times, and then withdrawn carefully to minimize contamination. Vaginal swabs were immediately placed in sterile Whirl Pak bags and transported on ice to the lab where they were transferred into 1 ml of BHI broth containing 20% glycerol and stored at − 80 °C until DNA extraction.

For uterine sampling, the reproductive tract was removed and immediately transported to the lab. After removal of adnexa (excess broad ligament, fat, etc.), 1 cm of the cranial aspect of the uterine horn was removed with a sterile scalpel. A double guarded culture swab (71 cm length swab, Reproduction Provisions L.L.C) was placed into the lumen of the uterine horn and guided through the horn into the uterine body. Once in the uterine body, the inner plastic portion and swab were extended to collect an uncontaminated sample, the swab was retracted into the inner sleeve, and both the inner and outer sleeves were removed from the uterus. The tip of the swab (approx. 2.5 cm) was then cut and placed into a sterile microfuge tube and immediately stored at − 80 °C until DNA extraction.

### Extraction of DNA from ruminal fluid, and nasopharyngeal, vaginal and uterine swabs

Total DNA from ruminal fluid samples was extracted using the Qiagen DNeasy PowerLyzer PowerSoil kit (Qiagen Inc., Germantown, MD, USA)^31^. Metagenomic DNA from the nasopharyngeal, vaginal, and uterine swabs were extracted using a Qiagen DNeasy Tissue kit (Qiagen Inc., Germantown, MD, USA) according to the manufacturer’s instructions with some modifications as outlined previously^31^. DNA was also extracted from environmental controls (room air swabs collected during vaginal and nasophayngeal sampling). Negative extraction controls were included for all extraction kits. The concentration of extracted DNA was measured using a NanoDrop ND-1000 spectrophotometer and PicoGreen assay. DNA was stored at − 20 °C until used for 16S rRNA gene sequencing.

### 16S rRNA gene sequencing and analysis

The V3-V4 hypervariable regions of the 16S rRNA gene were amplified and sequenced on a NovaSeq 6000 instrument (Illumina, San Diego, CA, USA) with a SP flow cell (2 × 250 bp) as previously described^31^. The 16S rRNA gene sequences were processed using DADA2 v. 1.20.0^32^ in R. 4.0.3 with the forward reads truncated at 225 bp and the reverse reads at 220 bp, merged with a minimum overlap of 20 bp, and amplicon sequence variants (ASVs) generated. Taxonomy was assigned to ASVs using the naïve Bayesian RDP classifier and the SILVA SSU release 138.1 database^33^. The ASVs that were classified as chloroplasts, eukaryota, or mitochondria were removed. Negative extraction and environmental (room air swabs) controls were also used to identify potential contaminants with ASVs removed if they had an abundance in a negative control that was equal or greater than the average abundance in a biological sample. The number of ASVs per sample (richness), the Shannon and inverse Simpson’s diversity indices, and Bray–Curtis dissimilarities were calculated in R using Phyloseq 1.38.0^34^ and vegan 2.5-7^35^. To account for uneven sequence depths, samples were randomly subsampled to 38,500, 6700, 27,000, and 4600 for the ruminal fluid, nasopharyngeal, vaginal, and uterine samples respectively, prior to the calculation of Bray–Curtis dissimilarities and alpha diversity measures.

### Statistical analysis

A linear mixed model using the lmer function in the lme4 v 1.1.27.1 R package^36^ was used to compare microbial diversity and richness measures by sampling time and diet. The linear mixed model included the random effect of the animal and the fixed effects of diet, sampling time, and their interactions as fixed effects. Post-hoc comparisons were performed within each sampling time and corrected for multiple comparisons using Tukey’s honestly significant difference. The effect of diet on the ruminal, nasopharyngeal, vaginal and uterine microbial community structures were assessed using the Bray–Curtis dissimilarities and PERMANOVA (adonis2 function) in R with vegan. The R package pairwiseAdonis v. 0.4^37^ was used to compare the Bray–Curtis dissimilarities within each sampling time for the nasopharyngeal and rumen samples, and the Benjamini–Hochberg procedure was used to correct *P* values for multiple comparisons. Differentially abundant genera between diet types were identified within each sample type using MaAsLin2 v. 1.8.0^38^ in R. Only those genera with a relative abundance of 0.1% or greater within the samples being assessed were included. The number of ASVs (richness), diversity indices, relative abundance of the most relatively abundant phyla in uterine samples between the two dietary groups were compared using the generalized liner mixed model estimation procedure (PROC GLIMMIX) in SAS (ver. 9.4, SAS Institute Inc., Cary, NC, United States). Statistical significance was considered at *P* < 0.05.

## Results

### Overview of the 16S rRNA gene sequencing

After processing and quality filtering, the average number of sequences per sample were 74, 360 ± 1275 (SEM), 50,233 ± 2574, 63,204 ± 3191 and 29,617 ± 8068, for the ruminal, nasopharyngeal, vaginal, and uterine samples, respectively. From these sequences, a total of 78,156 archaeal and bacterial ASVs were identified among all samples and classified into 34 phyla (one archaeal and 33 bacterial phyla) and 1432 unique genera.

### Effect of feeding hempseed cake on the ruminal microbiota

**Figure 2:**
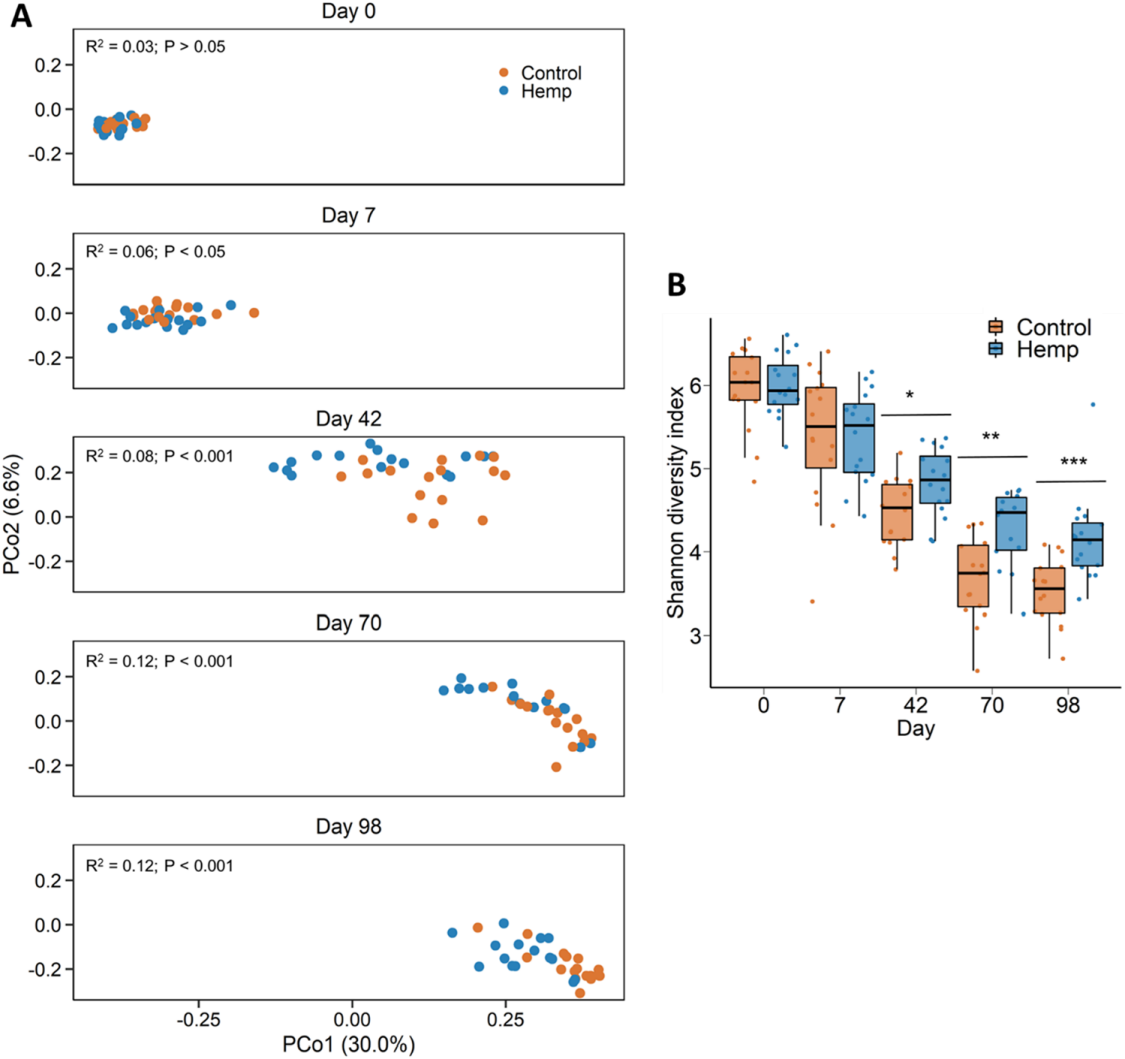
(**A**) Principal coordinates analysis (PCoA) plot of the Bray Curtis dissimilarities for the ruminal microbiota by diet and sampling day. The PERMANOVA R^2^ and P-value for the effect of diet within each day are included on each plot. The percentage of variation explained by each principal coordinate is indicated on the axes; (**B**) Box and whisker plot of the Shannon diversity index values for the ruminal microbiota by sampling day and diet. *P < 0.05; **P < 0.01; ***P < 0.001. The box indicates the interquartile range (IQR) (middle 50% of the data), the middle line represents the median value, and the whiskers represents 1.5 times the IQR; Control refers to the group of heifers that received DDGS in their diet (n = 15) and Hemp refers to the group that received the hempseed cake inclusion diet (n = 16).

**Figure 3:**
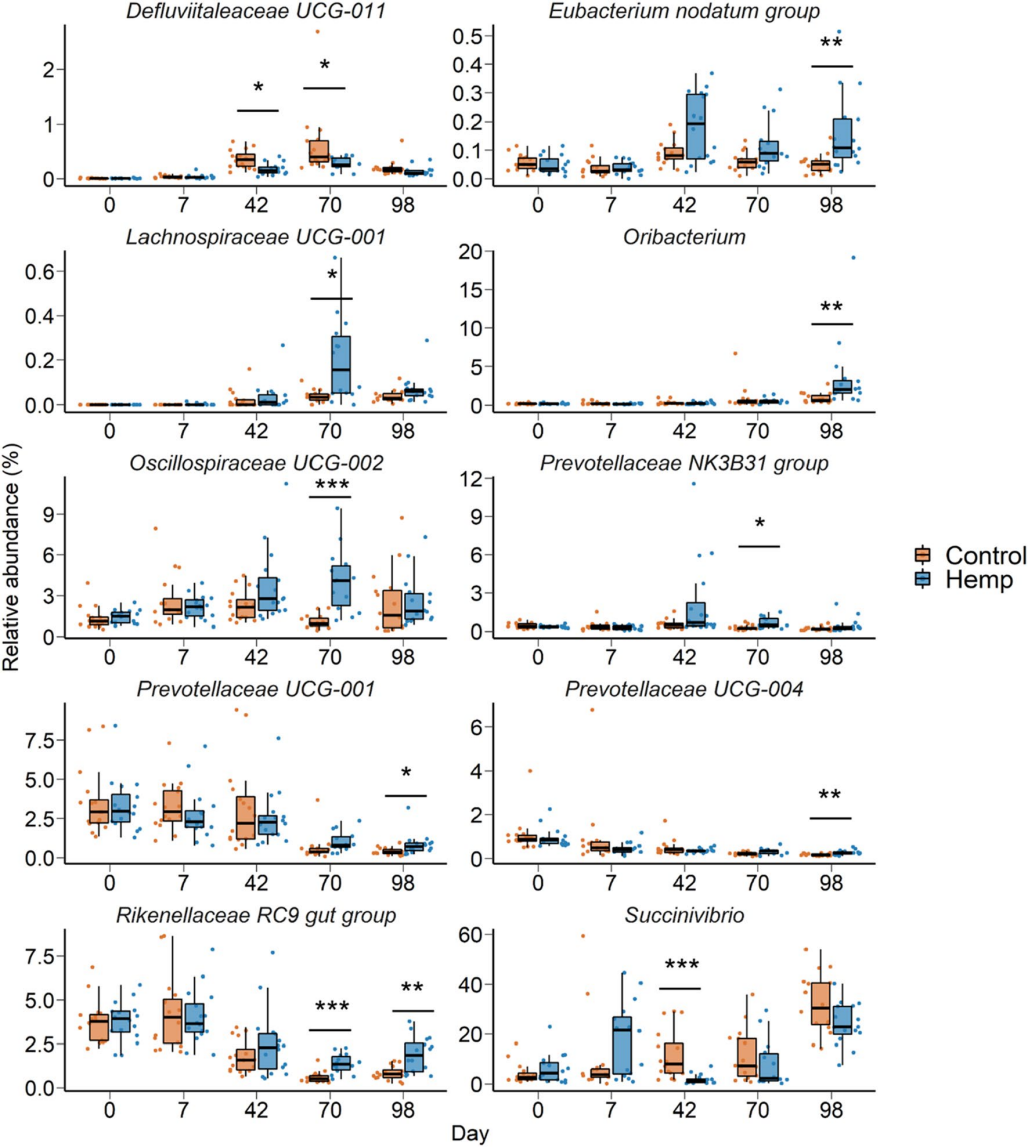
Box and whisker plots of the percent relative abundance of bacterial genera in the ruminal microbiota that were differentially abundant by dietary treatment on one or more sampling days. *P < 0.05; **P < 0.01; ***P < 0.001. The box indicates the interquartile range (IQR) (middle 50% of the data), the middle line represents the median value, and the whiskers represents 1.5 times the IQR; Control refers to the group of heifers that received DDGS in their diet (n = 15) and Hemp refers to the group that received the hempseed cake inclusion diet (n = 16).

Overall, there were 31 different bacterial (n = 30) and archaeal (n = 1) phyla detected from all the rumen fluid samples. Bacteria accounted for 99.19% and archaea 0.81% of the 16S rRNA gene sequences from the rumen samples. The dominant bacterial phyla included Bacteroidota (62.2%), Firmicutes (16.9%), Proteobacteria (16.3%), and Actinobacteriota (2.8%). The ruminal microbiota was significantly affected by sampling time during the study period (*R*^2^ = 0.39; *P* < 0.001). However, there were effects of diet on the ruminal microbiota structure from days 7 through to 98, with the greatest effect recorded on the last day of sampling, d 98 (*R*^2^ = 0.12; *P* < 0.001; Fig. 2A). Although microbial richness (number of ASVs) in the rumen was not affected by the inclusion of hempseed cake in the diet (*P* > 0.05), microbial diversity (Shannon diversity index) was greater in hempseed cake-fed cattle on d 42, 70, and 98 (Fig. 2B; *P* < 0.05). A number of bacterial genera in the rumen microbiota were differentially abundant between the control and hempseed cake diets starting on d 42 (Fig. 3). *Eubacterium nodatum* group, *Lachnospiraceae* UCG-002, *Oribacterium*, *Prevotellaceae* UCG-001, *Prevotellaceae* UCG-004, and *Rikenellaceae* RC9 gut group were among those genera enriched in the rumen microbiota of cattle fed hempseed cake. *Defluviitaleaceae* UCG-011 (d 42 and 70) and *Succinivibrio* (d 42) genera, however, were reduced in relative abundance in the hemp group compared to the control cattle.

### Effect of feeding hempseed cake on the nasopharyngeal microbiota

**Figure 4:**
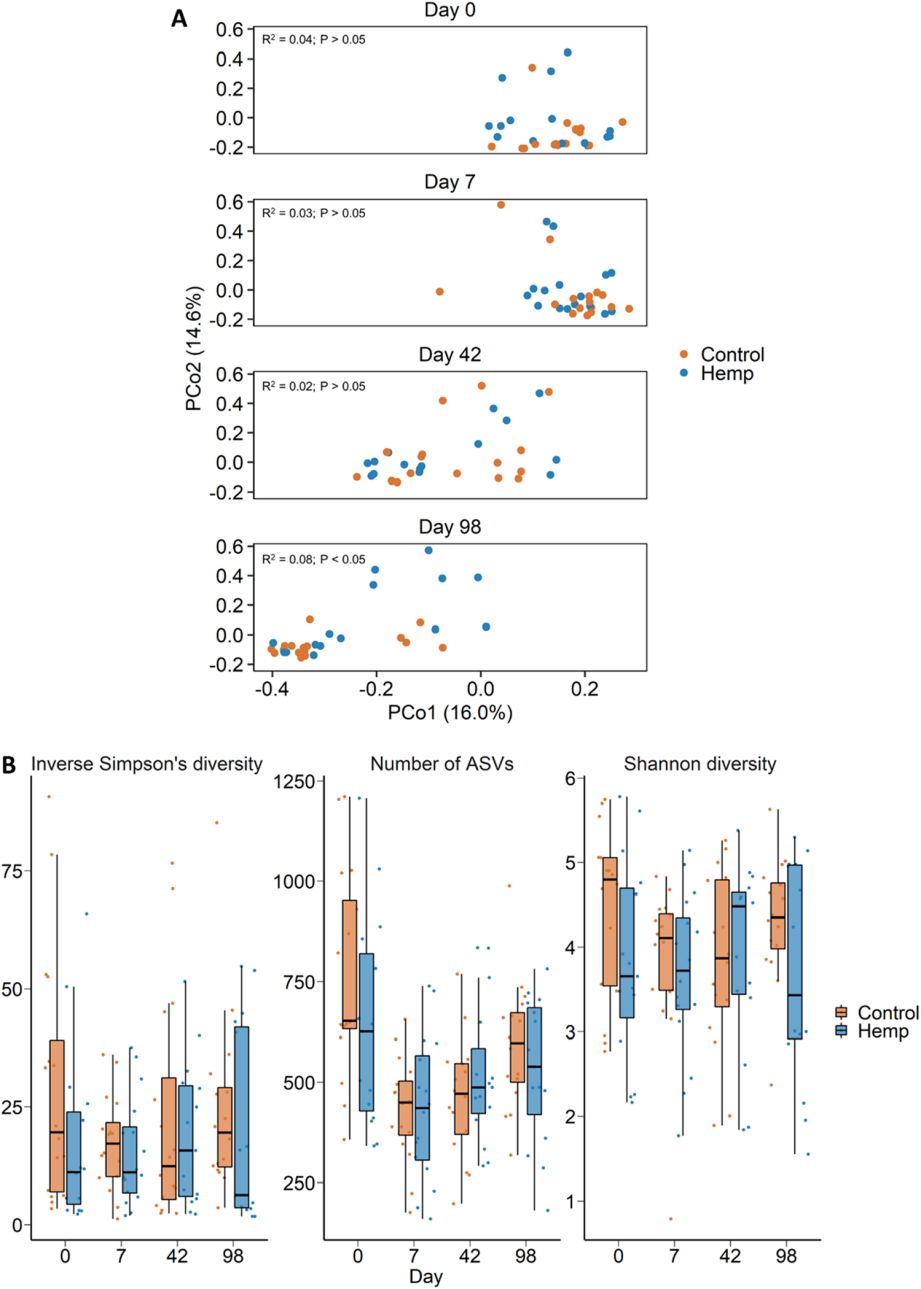
(**A**) Principal coordinates analysis (PCoA) plot of the Bray–Curtis dissimilarities for the nasopharyngeal microbiota by diet and sampling day. The PERMANOVA results for the effect of diet within each day is included on each plot. The percentage of variation explained by each principal coordinate is indicated on the axes; (**B**) Box and whisker plot of the number of ASVs and the Shannon and inverse Simpson’s diversity indices for the nasopharyngeal microbiota by sampling day and diet. The box indicates the interquartile range (IQR) (middle 50% of the data), the middle line represents the median value, and the whiskers represents 1.5 times the IQR; Control refers to the group of heifers that received 20% DDGS in their diet (n = 15) and Hemp refers to the group that received 20% hempseed cake inclusion diet (n = 16).

A total of 25 different bacterial (n = 24) and archaeal (n = 1) phyla were detected across the nasopharyngeal swabs. The nasopharyngeal microbiota was dominated by Actinobacteriota (41.7%), Firmicutes (30.8%), Bacteroidota (18.2%), Proteobacteria (5.2%), and Deinococcota (1.6%). Similar to the ruminal microbiota, sampling time had a greater effect on the nasopharyngeal microbiota structure (*R*^2^ = 0.18; *P* < 0.001; Fig. 4A) than the dietary treatment. Only on d 98 did hempseed cake in the diet have an effect on structure of the nasopharyngeal microbiota (Fig. 4; *R*^2^ = 0.08; *P* < 0.05) (Fig. 4A). Diet did not affect microbial richness and diversity in the nasopharynx (Fig. 4B; *P* > 0.05) and there were no significant differentially abundant genera between the two diet groups at any of the sampling times (*P* > 0.05).

### Effect of feeding hempseed cake on the reproductive microbiota

#### Vaginal microbiota

**Figure 5:**
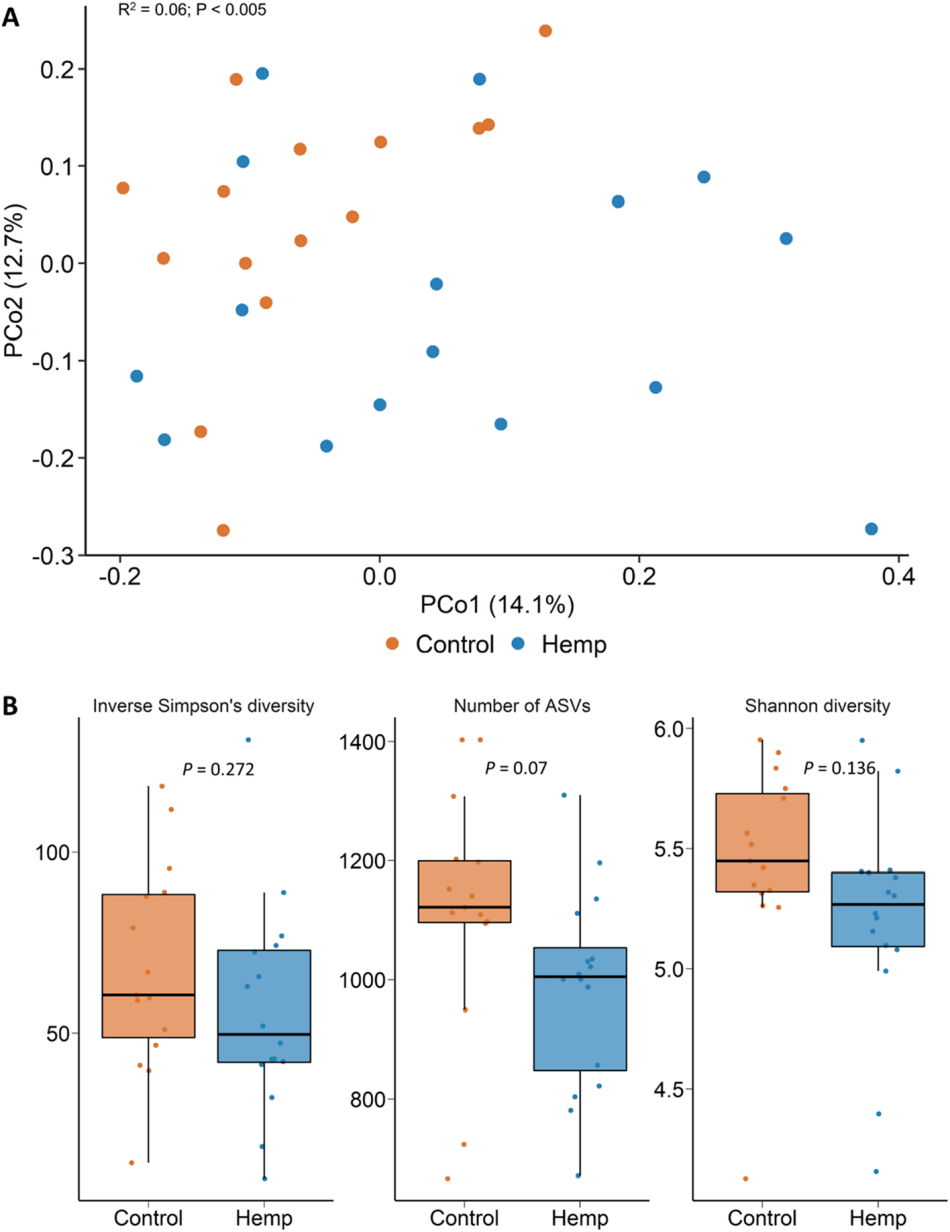
(**A**) Principal coordinates analysis (PCoA) plot of the Bray–Curtis dissimilarities for the vaginal microbiota by diet on d 112 (slaughter). The PERMANOVA result for the effect of diet is included on the plot. The percentage of variation explained by each principal coordinate is indicated on the axes; (**B**) Box and whisker plot of the number of ASVs and the Shannon and inverse Simpson’s diversity indices for the vaginal microbiota by diet on d 112 (slaughter). The box indicates the interquartile range (IQR) (middle 50% of the data), the middle line represents the median value, and the whiskers represents 1.5 times the IQR; Control refers to the group of heifers that received 20% DDGS in their diet (n = 15) and Hemp refers to the group that received 20% hempseed cake inclusion diet (n = 16).

**Figure 6:**
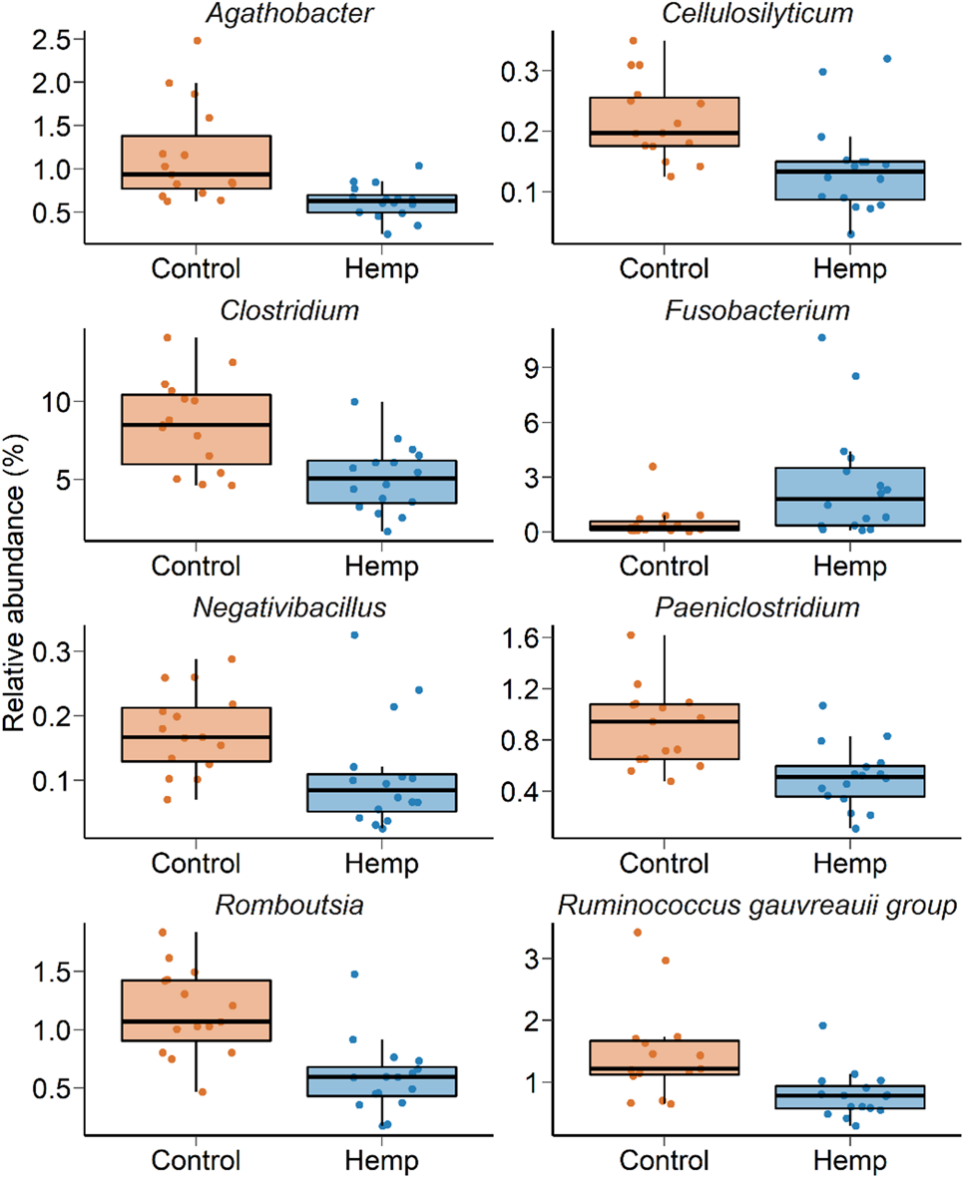
Box and whisker plots of the percent relative abundance of bacterial genera in the vaginal microbiota that were differentially abundant by dietary treatment (*P* < 0.05). The box indicates the interquartile range (IQR) (middle 50% of the data), the middle line represents the median value, and the whiskers represents 1.5 times the IQR; Control refers to the group of heifers that received 20% DDGS in their diet (n = 15) and Hemp refers to the group that received 20% hempseed cake inclusion diet (n = 16).

With the vaginal microbiota, there were 20 bacterial phyla and one archaeal phylum identified. The large majority of sequences belonged to the Firmicutes (53.5%), Bacteroidota (13.4%), Actinobacteriota (11.5%), Fusobacteriota (6.3%), Campylobacterota (4.8%), or Proteobacteria (4.6%) (Fig. 5A). Archaeal sequences accounted for only 0.05% of the total microbiota. On d 112 (at slaughter) there was a significant effect of diet on the vaginal microbial community structure (*R*^2^ = 0.06; *P* < 0.01) (Fig. 5A). While the microbial diversity measures did not differ between the two treatment groups (*P* > 0.05), hemp heifers tended (*P* = 0.07) to have lower microbial richness (total number of ASVs) compared to control heifers (Fig. 5B). Eight bacterial genera (*Agathobacter*, *Cellulosilyticum*, *Clostridium*, *Fusobacterium*, *Negativibacillus*, *Paeniclostridium*, *Romboutsia* and *Ruminococcus gauvreauii* group) were differentially abundant between the two diet groups (Fig. 6; *P* < 0.05). All but one of these genera, *Fusobacterium*, were more relatively abundant in the vaginal microbiota of the control cattle than in the hemp cattle.

#### Uterine microbiota

Only 8 uterine swab samples were included in the analysis (3 from hemp and 5 from control group) as the others did not pass the sequencing quality control, likely due to low microbial DNA concentration and/or low microbial biomass. A total of 22 bacterial and archaeal genera were detected across these 8 uterine swab samples. The uterine microbiota was mainly dominated by *Firmicutes* (34.1%), *Bacteroidota* (28.5%), *Proteobacteria* (21.4%), *Actinobacteriota* (14.2%), *Fusobacteriota* (0.6%), *Patescibacteria* (0.3%), *Campylobacterota* (0.2%) and *Cyanobacteria* (0.2%). The methanogenic archaeal phylum, *Euryarchaeota*, was also detected but only accounted for 0.05% of the total microbiota.

**Figure 7:**
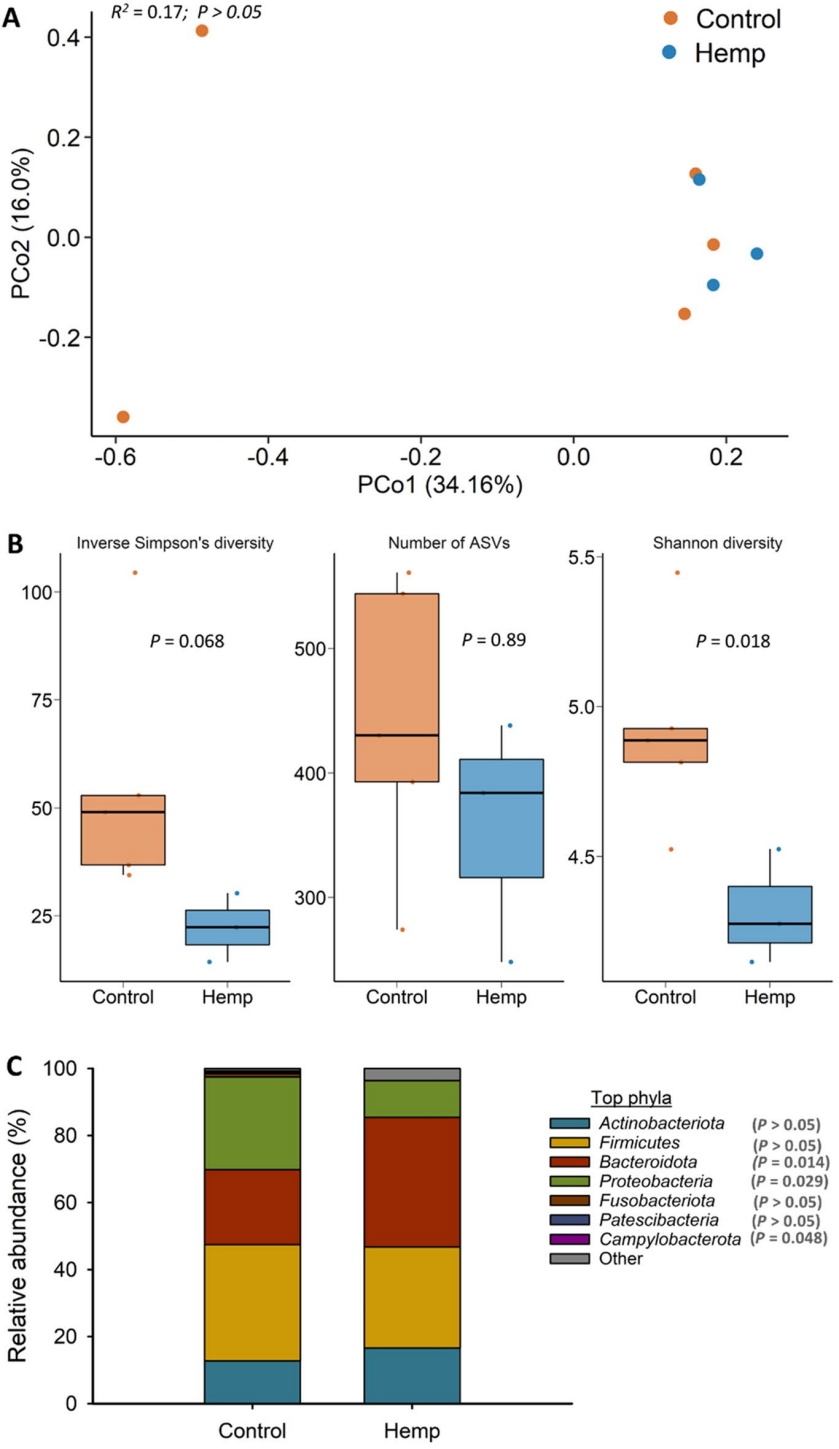
(**A**) Principal coordinates analysis (PCoA) plot of the Bray–Curtis dissimilarities of the uterine microbiota. The percentage of variation explained by each PCoA is indicated on the axes; (**B**) Box and whisker plot of the number of ASVs and the Shannon and inverse Simpson’s diversity indices for the uterine microbiota by diet on d 112 (slaughter). The box indicates the interquartile range (IQR) (middle 50% of the data), the middle line represents the median value, and the whiskers represents 1.5 times the IQR; (**C**) Stacked bar chart of the 7 most relatively abundant bacterial phyla in uterine microbiota by diet; Control refers to the group of heifers that received 20% DDGS in their diet (n = 5) and Hemp refers to the group that received 20% hempseed cake inclusion diet (n = 3).

Hempseed cake inclusion did not affect uterine microbiota community structure (*R*^2^ = 0.17; *P* > 0.05) (Fig. 7A) or microbial richness (*P* > 0.05) (Fig. 7B). Despite the sample size being small, significant differences were detected in microbial diversity between the two groups, with hemp heifers having lower Shannon diversity index values (*P* < 0.05) (Fig. 7B). The inverse Simpson’s diversity index also tended (*P* = 0.068) to be lower in the uterus of hemp heifers compared to control heifers. Hemp heifers had greater relative abundance of *Bacteroidota* (38.7% vs. 22.3%) and a reduced relative abundance of *Proteobacteria* (10.9% vs. 27.7%) and *Campylobacterota* (0.04% vs. 0.35%) compared to control group heifers (*P* < 0.05) (Fig. 7C).

### Similarities and core taxa shared across gastrointestinal, respiratory and reproductive tract microbiota

**Figure 8:**
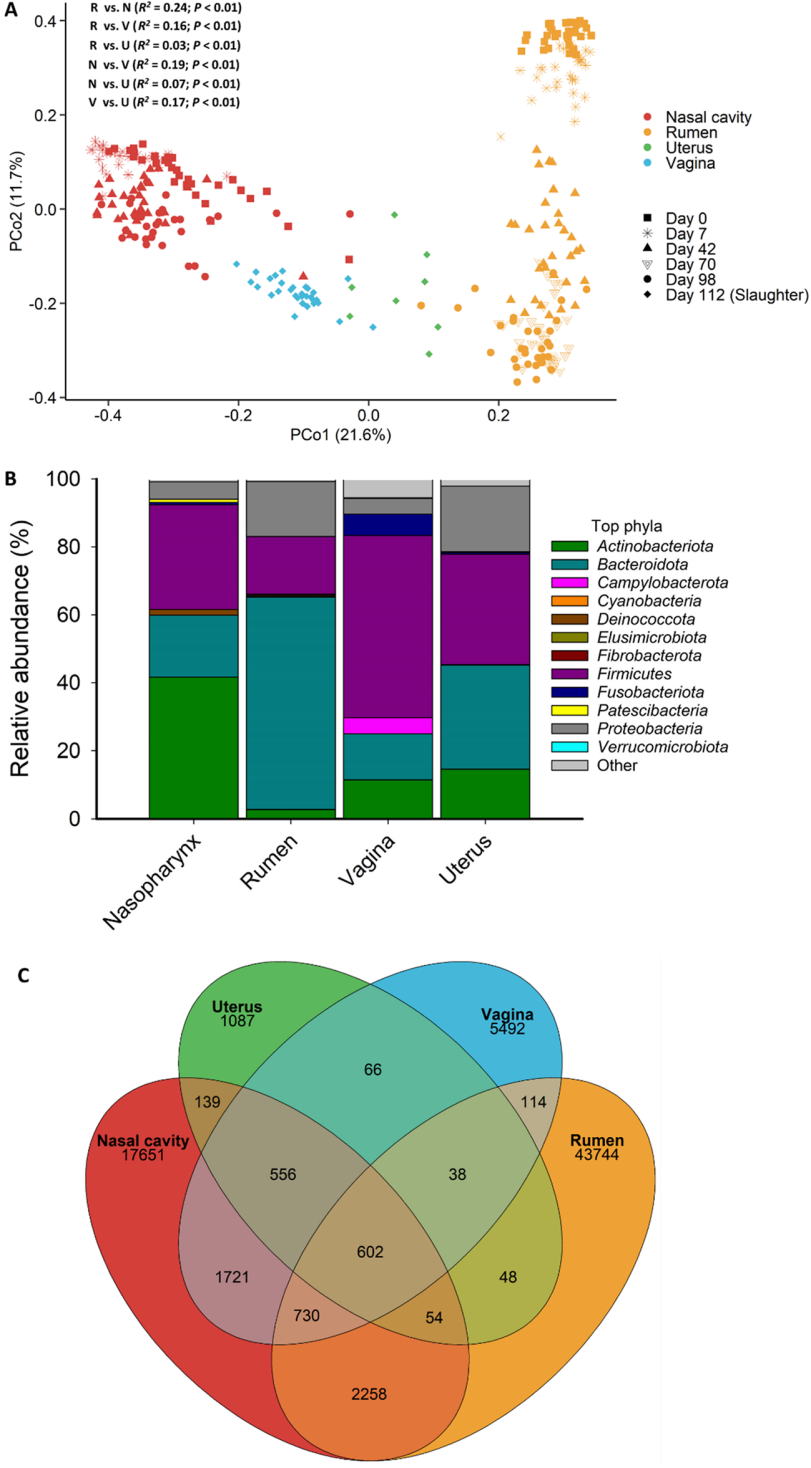
(**A**) Principal coordinates analysis (PCoA) plot of the Bray–Curtis dissimilarities of the nasopharyngeal, ruminal, vaginal and uterine microbiota by sampling day where applicable. The percentage of variation explained by each principal coordinate is indicated on the axes; (**B**) Stacked bar chart of the most relatively abundant bacterial phyla in these microbial communities; (**C**) Venn diagram displaying the number of shared and unique ASVs in the nasopharynx, ruminal, vaginal, and uterine microbiota independent of dietary treatment.Pairwise PERMANOVA comparisons are displayed in (**A**). R = rumen, N = nasopharynx, U = uterus, V = vagina.

We also compared the overall microbial community structure and composition and identified unique and shared ASVs among the ruminal fluid, nasopharyngeal, vaginal, and uterine swab samples (Fig. 8). As expected, the four sample types had distinct microbial community structures (PERMANOVA: *R*^2^ = 0.28; *P* < 0.001; Fig. 8A) with the ruminal and nasopharyngeal microbiota most dissimilar from each other (PERMANOVA: *R*^2^ = 0.24; *P* < 0.001) (Fig. 8A). Vaginal and uterine microbiota community structure were also significantly different from each other (PERMANOVA: *R*^2^ = 0.17; *P* < 0.001). Although 78,156 ASVs were identified among all samples, the vast majority of these were rare with only 602 ASVs found in at least one sample from each sample type (Fig. 8C).

**Figure 9:**
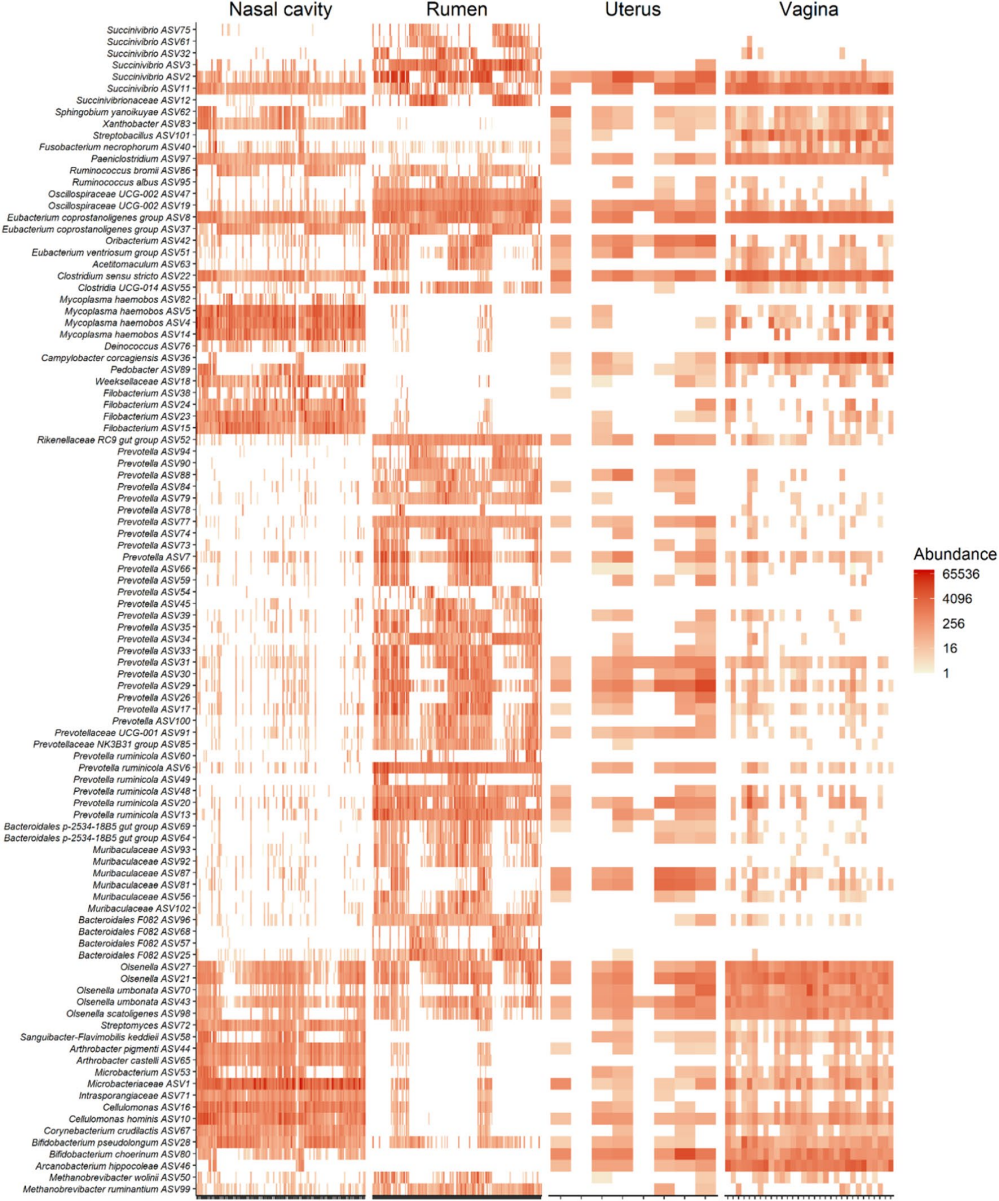
Heat map showing the 100 most abundant ASVs (log4) overall by sample type.

As shown in the heatmap of the 100 most abundant ASVs (Fig. 9), there was considerable inter-individual variation in both the prevalence and abundance of most the taxa present in ruminal, nasopharyngeal, vaginal, and uterine microbiota. Over two dozen ASVs within the *Prevotella* as well as several ASVs identified as *Prevotella ruminicola*, *Muribaculaceae*, and *Bacteroidales* were more frequently and abundantly found in the rumen, but they were mostly absent from the nasopharyngeal swabs. Some of these ASVs were present in vaginal and uterine swab samples but at lower frequency and abundance compared to the rumen fluid samples. ASVs identified as *Mycoplasma haemobos* and *Filobacterium* were mostly unique to the nasopharyngeal microbiota. ASV72 (*Streptomyces*), ASV44 and 65 (*Arthrobacter pigmenti*), ASV71 (*Intrasporangiaceae*), ASV10 (*Cellulomonas hominis*) and ASV67 (*Corynebacterium crudilactis*) were highly abundant in both the nasopharynx and vagina.

There were 28 ASVs that were found in at least 60% of all samples (Table 1). Six of these ASVs were also present in more than 80% of the samples. These included ASV8 (*Eubacterium coprostanoligenes* group; 99% of all samples), ASV197 (*Lachnospiraceae* NK3A20 group; 87%), ASV43 (*Olsenella umbonata*; 85%), ASV27 and 21 (*Olsenella* spp.), and ASV11 (*Succinivibrio* spp.).

**Table 1:**
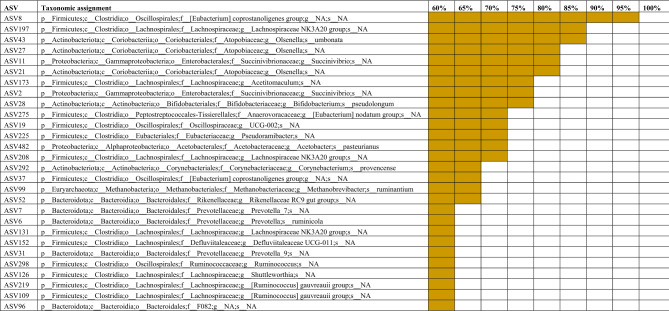
Core ASVs identified in at least 60% of ruminal fluid, nasopharyngeal, vaginal and uterine microbiota samples (N = 323) from finishing beef heifers.

## Discussion

We identified changes in the ruminal, nasopharyngeal, vaginal, and uterine microbiota in beef heifers fed a finishing diet containing 20% hempseed cake relative to changes in heifers fed 20% DDGS over the course of a 16-week feeding trial. All heifers appeared healthy throughout feeding trial and heifers fed hempseed cake consumed a similar amount of feed and exhibited similar feeding behaviours as heifers fed a DDGS^29^. However, hempseed cake inclusion led to a decrease in average daily gain (ADG), ultimately resulting in a lighter final body weight in hempseed cake-fed heifers than control heifers. Several factors may be contributing to the reduced ADG observed in the hemp group. As stated in our previous publication, this may include the greater acid detergent fiber concentration that was present in the hempseed cake compared to DDGS (16 vs. 11% on DM basis)^29^. Another factor associated with the lower feed to gain ratio observed in hempseed cake-fed heifers could be the altered ruminal microbiota and subsequent ruminal fermentation due to the ingestion of hempseed cake which contains anti-nutrient and antimicrobial agents^18,19,39^. This is evident by the significant alterations observed in the ruminal microbiota following hempseed cake feeding (Figs. 2 and 3).

Alterations in the ruminal microbiota was observed within a week of initiating hempseed cake ingestion despite the ruminal microbiota in these mature (19-month old) heifers being more resilient and robust compared to younger calves^40^. Starting from day 42 onward, the impact of hempseed cake ingestion on the ruminal microbiota became more evident as reflected by the significant differences in the microbial community structure, diversity, and composition between the hemp and DDGS-fed heifers (Figs. 2 and 3). Hempseed cake ingestion resulted in increased rumen microbial diversity (Shannon diversity index). Although alpha diversity metrics for the ruminal microbiota was reported to be the same between beef cattle with high or low feed efficiency^41,42^, the increased ruminal microbial diversity measured in the hemp group may be negatively associated with feed efficiency given the reduced ADG observed in hempseed cake-fed heifers. The compositional changes induced by hempseed cake ingestion were characterized by an increase in the relative abundance of six bacterial genera (*Eubacterium nodatum* group, *Lachnospiraceae* UCG-002, *Oribacterium*, *Prevotellaceae* UCG-001, *Prevotellaceae* UCG-004, and *Rikenellaceae* RC9); most of which have been reported to have associations (positive or negative) with feed efficiency in beef cattle^41–43^. For example, Liu and colleagues reported that there was a greater relative abundance of *Lachnospiraceae* in Angus heifers with low residual feed intake (RFI) (more feed efficient) compared with high RFI heifers^41^. Members of the *Rikenellaceae* RC9 gut group, *Lachnospiraceae,* and *Prevotellaceae* have also been reported to be present in greater abundance in ruminal fluid of Nellore steers with low feed efficiency compared to high feed efficient steers^42^. The low feed efficient Nellore steers also had greater abundance of *Succinivibrio* taxa than high feed efficient steers. Similarly, the relative abundance of *Rikenellaceae* RC9 gut group, *Prevotella* and *Succinivibrio* taxa were negatively associated with feed efficiency in Nellore beef cattle (male and female)^43^. Thus, the 10 genera (Fig. 3) that were differentially abundant in the rumen microbiota between the hemp and control cattle may be associated with reduced feed digestion and nutrient absorption given that most of these genera are known to be associated with feed efficiency in cattle. Therefore, further research is warranted to evaluate the effect of hempseed cake inclusion in the diet on the members of these genera, and fermentation parameters in vitro.

The significant alteration in ruminal microbiota community structure, diversity and composition observed in hemp heifers may be attributed to several antimicrobial components contained within the hempseed cake. The oil content (7%) may have influenced the ruminal microbial composition as the antibacterial activity of hempseed oil against a wide range of Gram-positive bacterial species has been well documented^18,44,45^. In addition, the psychoactive components and cannabinoid derivatives in hempseed cake, have known antimicrobial activity against a large panel of Gram-positive and Gram-negative pathogens^46^, and may have similar activity in the rumen. Overall, the results of our study indicate the need for further research to investigate the impact of feeding hemp by-products on the gut microbiota and fermentation parameters.

Few effects of feeding hempseed cake were observed in the upper respiratory tract microbiota. Only on day 98 was the community structure of the nasopharyngeal microbiota significantly different between the hemp and DDGS fed heifers. This is a particularly interesting observation as the impact of diet on the respiratory microbiota in cattle has infrequently been investigated. Hall and colleagues reported that feeding selenium-biofortified alfalfa hay for 9 weeks resulted in an altered nasopharyngeal microbiota in weaned beef calves^47,48^. Vitamin and mineral supplementation during the first 6 months of gestation has also been reported to induce some compositional changes in the nasopharyngeal microbiota of pregnant beef heifers^31^. Our results here suggest that longer feeding periods may be required to observe dietary alterations of the bovine respiratory tract microbiota in response to diet.

The microbiome-gut-lung axis may be a potential mechanism through which dietary inclusion of hempseed cake may influence the respiratory microbiota^49,50^. It is also likely that changes in the composition of the rumen gas phase, and potentially the microbes associated with ruminal gas because of hempseed cake ingestion may have affected the respiratory tract microbiota given that about 70–85% of the eructed gases from the rumen are inhaled^51^. Thus, considering the role of the respiratory microbiota in maintaining respiratory health and resilience against bovine respiratory disease, the costliest disease in the modern finishing cattle industry^52–54^, the long-term impact of feeding hempseed by-products on the respiratory microbiota and pulmonary health of cattle should be investigated.

Dietary hempseed cake not only affected the gastrointestinal and respiratory tract microbiota but also the microbiota in the reproductive tract, as we observed changes in both the vaginal and uterine microbiota. The vaginal microbiota at slaughter (d 112) in heifers fed hempseed cake was distinct in terms of community structure, microbial richness, and composition in comparison with the control heifers. Microbial richness in the vagina was reduced in the hemp group. In humans, reduced richness in the vaginal microbiota has been reported to be positively associated with reproductive health and pregnancy while increased microbial richness in the vagina was found in women who experienced preterm birth^55^ or reproductive infection (bacterial vaginosis)^56,57^. Thus, the observed decrease in microbial richness in the vagina associated with hempseed cake consumption could be an indicator of a healthy restructuring taking place within the vaginal microbiota. However, the compositional changes observed in the vaginal microbiota at the genus level suggest otherwise as shown by the reduced relative abundance of the most predominant and commensal vaginal genera such as *Clostridium*^58,59^ and *Romboutsia*^31^. Conversely, an elevated relative abundance of potentially pathogenic *Fusobacterium* spp. in heifers fed hempseed cake was observed*.*

The *Fusobacterium* genus includes the pathogenic species *Fusobacterium necrophorum,* and *Fusobacterium nucleatum* that are often involved in reproductive infections and abortions, as well as liver abscesses in cattle^60–63^. Among the other bacterial genera whose relative abundance was depleted in the vaginal microbiota of hemp heifers, *Agathobacter*, *Negativibacillus* and the *Ruminococcus gauvreauii* group are largely commensal genera that are also dominant members of the bovine^64,65^ and human gut microbiota^66^. The genus *Paeniclostridium* which contains the pathogenic species *Paeniclostridium sordellii *(previously known as *Clostridium sordellii*), is associated with “sudden death syndrome’ in feedlot cattle^67^, septic shock in women^68^ and enterocolitis in horses^69^. Taken together, however, the observed changes in the vaginal microbiota of hempseed cake-fed heifers are insufficient to make conclusive statements regarding a positive or negative influence of hempseed cake consumption on the vaginal microbiota of heifers. Future studies are needed to verify the impact of hempseed by-product on reproductive microbial composition, particularly on pathogenic species (*Trueperella pyogenes*, *F. necrophorum, F. nucleatum* and *P. sordellii*), and reproductive health and fertility in female cattle.

The alterations observed in the vaginal and uterine microbiota by hempseed cake intake may be because of several factors. First and most importantly, hempseed cake supplementation induced alterations in microbial population and fermentation parameters in the gut (rumen) that may lead to an altered metabolite profile (e.g. short-chain fatty acids and immunomodulators) in the blood and/or peripheral blood mononuclear cells associated with specific bacteria^70^. Consequently, this may influence the microbial communities along the reproductive tract^50^. Second, hempseed cake ingestion may have affected the production of reproductive hormones, which are known to shape the vaginal microbiota^71,72^. Third, similar to the respiratory microbiome, the microbiome-gut-reproductive axis may be associated with changes in the reproductive tract microbiome due to hempseed cake consumption^50^. Finally, cannabinoid derivatives in hempseed cake may have altered the immune cell and cytokine profiles in the reproductive tract given the well-documented immunomodulatory properties of cannabinoids^73^.

Overall, the results of our study suggest that consumption of hempseed cake can influence the female reproductive microbiome. Also, given that the heifers used in our study were approximately 21 months of age when sampled for vaginal and uterine swabs, the impact of feeding hempseed by-product on the genital microbiota would be expected to be stronger in younger female cattle as they typically have a less robust and resilient reproductive microbiota compared to older cattle^74^). Including hempseed cake in the diet of female cattle used for breeding may impact reproductive microbiome-mediated fertility and pregnancy outcomes. Therefore, more attention should also be given to investigate the impact of feeding hemp by-products in cow-calf operations.

The gastrointestinal tract microbiome is most often the target of dietary interventional studies in both livestock and humans as it is the largest microbial community in the host and plays a central role in host nutrient metabolism and health. Research on the respiratory and reproductive tract microbiome are more frequently focused on infectious microorganisms and diseases as nutrient interventions have been thought to have less impact on these extra-gut microbial ecosystems. However, recent evidence suggests that gut microbiome-mediated health may rely on communication with different organs throughout the body (e.g. lung/reproductive/mammary)^50^. It has also been suggested that the gut microbiome affects distant organs and host metabolic pathways through mediation of endocrine systems^75^. In addition, many bacterial taxa present in the digestive tract are also present in the respiratory and reproductive tracts^31,75^. Accordingly, we hypothesized that changes in the gut microbiome due to dietary treatment may also affect microbial communities in other organs.

Therefore, we evaluated the impact of hempseed cake inclusion in the diet on not only the ruminal microbiota but also on the respiratory (nasopharyngeal), and reproductive (vaginal, and uterine) microbiota. The alterations observed here in these four microbial communities in response to hempseed cake feeding support the existence of interconnection among these microbiota, potentially through mechanisms involved in the microbiome-gut-respiratory and microbiome-gut-reproductive axes^50^. As expected, the composition, diversity, and structure of the ruminal, nasopharyngeal, vaginal, and uterine microbiota were significantly different from each other due to the physiological and anatomical differences in the mucosal surfaces of these anatomical sites^31^. Although the rumen, nasopharynx, vagina, and uterus have drastically different physiological and anatomical properties, we identified that 28 ASVs were shared by a relatively high proportion (60%) of all samples. Thus, these “core taxa” may be involved in facilitating communication among the gut, respiratory and reproductive microbial communities or could colonize multiple host sites.

Some of the observations reported in this study can potentially be extrapolated to humans as cattle have similar physiological and developmental characteristics and are colonized by microbes that are biogeographically and phylogenetically more similar to those found in the human microbiota when compared with rodent models^28^. The alterations seen here in the bovine gut microbiota, respiratory, and reproductive microbiota due to hempseed cake feeding suggest that the effect of hemp products on the human microbiome and health should also be investigated. In particular, the impact of hempseed cake feeding on the vaginal and uterine microbiota that we observed highlights the need for future research into the impact of feeding hempseed oil and other hemp products on fertility and reproductive health in women.

### Strengths and limitations of the study and future direction

There were several strengths and limitations in our study. One of the strengths to our study is associated with the longitudinal sampling of rumen fluid and nasopharyngeal swabs, which allowed us to detect any changes in ruminal and nasopharyngeal microbiota that occurred over a period of 98 days in response to hempseed cake feeding. Another strength lies in the holistic approach that we applied to evaluate the impact of feeding hempseed by-product not only on the gut microbiota but also on the microbial communities associated with the respiratory and reproductive tracts by sampling four different locations from a single animal. Finally, the data regarding hempseed product ingestion and microbiome that we generated in this study are from bovine animals, and thus have some implications for directing research on hemp product consumption and human microbiome.

Although the rumen and nasopharyngeal microbiota were sampled multiple times, the vaginal swabs were collected only at the end of the feeding trial. For logistical reasons, the vaginal samples had to be done over 4 days, which may have increased inter-individual variation. In addition, only 8 out of 31 uterine swabs collected at necropsy could be sequenced and included in this study. Therefore, the results of uterine microbiota need to be interpreted cautiously. Uterine swabbing of live cattle, especially in virgin yearling heifers, can be invasive, and makes it difficult to collect samples over multiple time points. Nonetheless, we were able to detect differences in the vaginal and uterine microbiota between the two treatment groups. Future studies are warranted to assess the longitudinal impact of feeding hempseed cake on both taxonomic and functional characteristics of bovine reproductive microbiome. Also, building on the results on the impact of hempseed cake on ruminal microbiota composition and community structure, how the ingestion of hempseed cake can influence the function of ruminal microbiome, ruminal fermentation parameters, as well as microbial community along the hindgut should be further investigated using shotgun metagenomic, metabolomic and in vitro techniques.

## Conclusion

In summary, sampling time had a significant effect on both ruminal and nasopharyngeal microbial community structure. Hempseed cake feeding resulted in significant alterations in the ruminal microbiota starting from day 7 through to day 98, including a distinct community structure, increased Shannon diversity index values, and enrichment of eight bacterial genera. Hempseed cake had a smaller effect on the nasopharyngeal microbiota, but the microbial community structure of the two dietary treatment groups cattle differed on day 98. The vaginal and uterine microbiota community structure, richness, and composition was also affected by hempseed cake. Additionally, we identified a small set of core taxa that were shared among the different sample types. Overall, the results of our longitudinal study suggest that feeding hemp by-products can alter the bovine gut, respiratory and reproductive microbiota. This finding indicate that future research aiming to evaluate the use of hemp by-products in livestock diet should consider their impact on animal microbiome and microbiome mediated animal health and reproductive efficiency. The findings of this study also highlight the need for research evaluating the impact of hemp-associated food and personal care products on the human gut, respiratory and reproductive microbiome.

## Data Availability

Raw sequence data are available from the NCBI Sequence Read Archive under BioProject accession PRJNA838018 (https://www.ncbi.nlm.nih.gov/bioproject/?term=PRJNA838018). Other data that supports the findings of this study are presented within the paper.
